# Networks of stress, affect and eating behaviour: anticipated stress coping predicts goal-congruent eating in young adults

**DOI:** 10.1186/s12966-020-01066-8

**Published:** 2021-01-09

**Authors:** Björn Pannicke, Tim Kaiser, Julia Reichenberger, Jens Blechert

**Affiliations:** 1 Department of Psychology, Centre for Cognitive Neuroscience, Paris-Lodron-University of Salzburg, Salzburg, Austria; 2 Department of Psychology, Clinical Psychology and Psychotherapy, University of Greifswald, Greifswald, Germany

**Keywords:** Network analysis, mlVAR, Stress coping, Affect, Healthy eating, Goal-congruent, Hunger, Food craving, Diet

## Abstract

**Background:**

Many people aim to eat healthily. Yet, affluent food environments encourage consumption of energy dense and nutrient-poor foods, making it difficult to accomplish individual goals such as maintaining a healthy diet and weight. Moreover, goal-congruent eating might be influenced by affects, stress and intense food cravings and might also impinge on these in turn. Directionality and interrelations of these variables are currently unclear, which impedes targeted intervention. Psychological network models offer an exploratory approach that might be helpful to identify unique associations between numerous variables as well as their directionality when based on longitudinal time-series data.

**Methods:**

Across 14 days, 84 diet-interested participants (age range: 18–38 years, 85.7% female, mostly recruited via universities) reported their momentary states as well as retrospective eating episodes four times a day. We used multilevel vector autoregressive network models based on ecological momentary assessment data of momentary affects, perceived stress and stress coping, hunger, food craving as well as goal-congruent eating behaviour.

**Results:**

Neither of the momentary measures of stress (experience of stress or stress coping), momentary affects or craving uniquely predicted goal-congruent eating. Yet, temporal effects indicated that higher anticipated stress coping predicted subsequent goal-congruent eating. Thus, the more confident participants were in their coping with upcoming challenges, the more they ate in line with their goals.

**Conclusion:**

Most eating behaviour interventions focus on hunger and craving alongside negative and positive affect, thereby overlooking additional important variables like stress coping. Furthermore, self-regulation of eating behaviours seems to be represented by how much someone perceives a particular eating episode as matching their individual eating goal. To conclude, stress coping might be a potential novel intervention target for eating related Just-In-Time Adaptive Interventions in the context of intensive longitudinal assessment.

**Supplementary Information:**

The online version contains supplementary material available at 10.1186/s12966-020-01066-8.

## Background

Adhering to a healthy diet − as for instance recommended by the World Health Organization [1] − benefits cardiovascular, metabolic and mental health [2–4], greater longevity [5] and also helps in maintaining a healthy weight [6]. As a result, many individuals monitor and manage their eating to some degree, for example by using smartphone apps [7], and follow a more or less explicit goal to eat healthily. However, as specific eating goals might vary notably between individuals and dietary interventions can be rather heterogeneous [8], it can be difficult to define healthy eating (behaviours) in absolute terms. Thus, deviations from personal goals are a topic of intense research in the domain of self-regulation [9]. Undoubtedly, adhering to personal goals in eating (i.e. ‘goal-congruent eating’) requires self-control [10–13], given external factors such as the omnipresence of tempting unhealthy foods in today’s affluent environments [14–16]. The hedonic appeal of such foods can cause unhealthy food consumption [17, 18] and might thereby threaten goal pursuit with regard to healthy eating. Yet, both self-control and healthy eating can also be threatened by internal factors such as food cravings [19], stress experience [20], coping (such as *avoidance coping* [21]) and affects (such as *negative urgency* [22]), each of which will be reviewed in the following.

Firstly, *food cravings* can interfere with goal-congruent eating and can threaten diet adherence [23]. Unlike hunger – which can be satisfied by any type of food – *food cravings* represent urges to eat specific foods [24] that are often high in palatability. Chocolate and other high-sugar and high-fat snacks are the most frequently craved types of food [19, 25, 26]. Still, strong food cravings do not necessarily lead to food consumption, due to a number of intermittent variables like social context and food availability, however, such cravings often precede consumption [19, 27] and can predict snack consumption better than hunger [19]. Thus, the occurrence of food cravings might be predictive for subsequent less goal-congruent eating behaviours.

Secondly, *stress* interferes with self-control and thus influences eating behaviours [20]. According to Lazarus and Folkman [28], stress can be described as a cascade of a stressor triggering a primary appraisal (i.e. interpretation of the stressor), followed by a secondary appraisal (i.e. analysis of the available resources). If a stressor is interpreted as threatening and resources seem insufficient, an individual will experience stress. According to this theory, reactions to such stress experience can be either problem-orientated or emotion-orientated coping [28]. Yet, there are also other stress theories [29] that cannot be reviewed in detail in the present paper. To exemplify with regard to eating behaviours, physiological accounts attribute the relationships between stress and eating behaviours to the elevated energy needs implied in the response to stressors [30]. Increased stress-related food intake has been linked with activity of the hypothalamic-pituitary-adrenal axis [31] with cortisol as the central metabolic regulator [32, 33]. Importantly, the direction of relationships between stress and eating can vary considerably between individuals [34, 35]: While some studies found that stress increased the amount of consumed foods in real-life environments [36] and laboratory settings [37], others found that stress reduced the amount of subsequent food consumption [38, 39]. Such heterogeneity points to the role of individual difference variables such as learned stress-eating patterns [40] as well as stress coping. Besides the experience of stress, coping styles were also related to eating behaviours in several studies: Previous research reported associations of both emotion-orientated and avoidance coping with eating in response to affects [41], with eating disturbances [42] as well as with binge eating [43].

Thirdly, *affects* – i.e. subjective experiences of certain moods [44] *–* can also influence eating behaviour and thereby contribute to whether personal eating goals are met: snacking of unhealthy but palatable foods might reduce negative affects [18, 37, 45, 46] and can therefore be reinforced through operant learning processes [47]. Similar to the relationships between stress and eating, individual differences characterise the linkage between affects and eating: some affects (for instance *sadness*) are reported to increase the intake, while others (for instance *anger*) are reported to decrease it [35, 48]. On the other hand, also positive affects can lead to increased food intake or snacking frequency [49]. Such findings are often discussed in the light of the individual difference model of emotional eating [50], which states that individuals can have between-person characteristics that influence how they react to stress or affects with regard to eating. As influences of affects on eating behaviours might be especially relevant in the context of weight control, emotional eating is also discussed as a potential mediator for weight gain in dieting females [51]. In general, varying findings regarding affects and eating behaviours contribute to a lively debate about the existence and nature of emotional eating [37, 52–56].

**Fig. 1:**
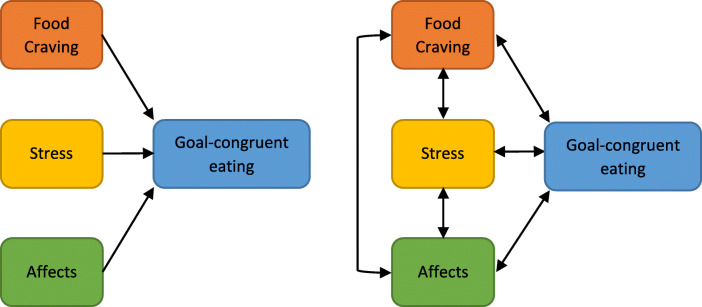
**a** Simplified, unidirectional conceptual model. **b** Multidirectional conceptual model

The multitude of factors that seem to influence goal-congruent eating call for a multivariate analytic approach. Figure 1a summarises potential unidirectional predictive relationships of craving, stress and affects on goal-congruent eating. Yet, the ‘predictor variables’ craving, stress, and affects also influence each other, casting doubt on the simple model in Fig. 1a. Intuitively, more stress can be associated with higher negative affect [57] as well as more craving [40] in daily life. Additionally, affects (and their regulation) were found to be related to food cravings [24]. Hence, the influence of these variables on eating behaviour might also be indirect − e.g. affect mediates between stress and eating behaviour [58] − or potentially interactive. Moreover, eating behaviour might in turn influence subsequent stress related and affective experiences [59, 60], leading to a reversed direction in the causality assumed in the simplified model. Thus, associations between stress/affect/craving and eating behaviour are likely multidirectional through feed-forward (i.e. ‘predictors’ influence eating behaviour) and feedback (i.e. eating behaviour influences ‘initial predictors’) loops that are illustrated in Fig. 1b.

The potential presence of multiple pathways and causal directions has implications for assessments and analytical procedures. Cross-sectional, single-wave questionnaire studies are unable to fulfil these conditions. While experimental research in the laboratory might establish causality, it is limited in mapping naturalistic variations in eating behaviour, stress and affect in everyday life due to the artificial context and effects of observation [61, 62]. Ecological momentary assessments (EMA) allow for a naturalistic investigation [63] of concurrent and prospective (directed) associations between stress, affect and craving on the one hand and eating behaviour on the other as highlighted in Fig. 1a. Multilevel vector autoregressive network analyses – a rather recent extension to the classical, multilevel modelling / regression based statistical approach – allow for a simultaneous assessment of cross-links [64] between the potential predictor variables stress, affect and food craving. Thus, network analyses are also able to model feedback (loops) between variables that are related among each other [64]. Based on longitudinal time-series data, network models can answer directionality questions such as whether *negative* affects influence eating behaviour and / or vice versa. Such network models can also determine which variables might be most important in a multidirectional structure around eating behaviour [64]. In the following, we will firstly elaborate on the characteristics of a network analysis before describing its implementation in the present study context as well as our research questions.

### Multilevel vector autoregressive networks

For examining unique associations between variables (i.e. by controlling for all other effects in a model) and further illustrating Gaussian graphical models (GGM) based on regularised partial correlations, two types of data are of interest: cross-sectional data (i.e. variables measured on one occasion in many subjects) and time-series data (i.e. variables measured on several occasions) [65]. Suppose the given data is based on multiple measurements of at least one participant (i.e. (multivariate) time-series), vector autoregressive (VAR) modelling can be conducted allowing to model two network structures, i.e. a temporal network based on repeated measures and a contemporaneous network obtained through GGM based models of the VAR’s residual structure [66]. Additionally, if time-series data of *N* > 1 are analysed, a between-subject network can be modelled through applying the GGM to the covariance structure of stationary means [66]. Combining modelling of time-series data with cross-sectional analyses, multilevel vector autoregressive (mlVAR) analyses allow to visualise multidirectional relationships in three different networks in which ‘nodes’ (i.e. all variables included in the analysis) are linked by ‘edges’ (i.e. the relationships between two variables). Firstly, *temporal networks* predict each node within individuals by all other nodes at the previous time point as well as by its own previous value (i.e. autocorrelation), thus representing directed (time-lagged) partial correlations based on vector autoregressive analyses of time-series data [64]. Secondly, *between-subject* networks illustrate the mean mutual regression of all nodes in form of a cross-sectional analysis. An edge that connects two nodes in a between-subject network represents the mean of two regressions that are calculated using both nodes once as predictor and once as outcome. In this way, between-subject networks can be interpreted as cross-sectional associations between variables over the assessment period (e.g. ‘*People who often report high values on x, often report high values on y, too.*’). Yet, this network summarises all moments within persons regardless of when values co-occurred. Thus, thirdly, *contemporaneous networks* allow displaying associations of simultaneous data. Technically, these associations are partial correlations representing the residuals after estimating both previous models. The contemporaneous network analysis complements the others and allows statements as ‘*X and y often occur simultaneously during the assessment period’* (for details, see [66, 67]).

In the present study, we utilise an entirely data-driven method, representing a rather novel approach in eating behaviour EMA research. Because of the potential multidirectionality of associations between all the variables described above it is difficult to state firm, directed hypotheses. Yet, most of the research mentioned above suggests that goal-congruent eating might be related to (or as a potential dependent variable influenced by) craving, stress and / or affects in one way or another (i.e. negatively or positively associated). Moreover, differences between *homeostatic* (‘hunger’ ratings) and *hedonic* eating behaviour (‘craving’ ratings) should emerge [68], particularly in their relationships with stress and affect variables [53]. Furthermore, feedback loops between nodes in the temporal network would be of particular interest, as they may indicate mutual amplification processes (e.g. experiencing stress increases eating, which in turn increases the experience of stress). Likewise, as indicated above, coping with stress relative to experiencing stress might reveal differential associations (for instance because of different appraisals). We also expect a clustering of positive affect items as well as of negative affect items, as they are often averaged to calculate scores. However, based on the literature reviewed above and given that the present analyses have – to our knowledge – not been conducted before in the field of healthy eating behaviour research, the concrete relationships between affect, stress and eating related variables are difficult to predict.

## Methods

### Participants

Individuals were recruited by means of a study announcement via e-mail and by word of mouth at several universities across Austria and Germany. Initially, data of 90 participants were collected. In the beginning, participants were asked to answer an entry questionnaire regarding their individual diet goal. The following two questions inquired whether individuals had interest in maintaining or reducing their current body weight: 1) ‘*Do you currently pay attention to your nutrition in order to maintain or reduce your body weight’? *and 2) ‘*Do you currently cut down on your food intake in order to maintain or reduce your body weight’?* To take part in the study it was required to agree to at least one of the two questions, thus, to be currently diet-interested. Out of 90 participants, 6 participants were later excluded because they answered less than 50% of all EMA questionnaires. Thus, 84 individuals were included in the network analyses. Because of missing descriptive data for one participant included in the analyses, descriptive statistics are based on 83 individuals. All participants received written as well as oral information on the purpose of the study and signed an informed consent according to the relevant ethics committee at the University of Salzburg, Austria, that also granted ethical approval. The signed consent form was either manually handed or electronically sent via e-mail to the research team.

### Procedure

If suitable for participation, individuals received several questionnaires (e.g. general eating behaviour styles; not of relevance for the present study) to answer electronically via the online platform LimeSurvey [69] and self-reported their current body weight and height, which were later used to calculate the BMI, as well as (if applicable) previous or current eating disorders. After that, participants were instructed how to use the smartphone app (‘*PsyDiary*’), which was collaboratively designed with the SmartHealthCheck work group from the department of MultiMedia Technology of the University of Applied Sciences Salzburg (FH Salzburg, Austria). PsyDiary draws on LimeSurvey for creating EMA questionnaires, runs on Android or iOS systems, and can be installed via the local app store. The smartphone app has previously been used in other studies [19, 53, 68]. The app prompted participants for 14 consecutive days asking them to answer questions regarding current states as well as recently consumed foods. At the beginning of the EMA, participants were given an additional practice day to get used to the app (data not used in the present study). During EMA data assessment, compliance (i.e. completion rate regarding EMA questionnaires) was continuously monitored to ensure a rather high response rate. In case of an accumulation of missed or unanswered EMA questionnaires, we contacted participants to inquire whether technical or other issues occurred. After the two-week period, participants received final questionnaires that included items concerning compliance and reactivity. Subjective compliance was assessed by the question *‘How often did you try to shorten EMA questionnaires by purposefully answering questions in such a manner that no additional questions appeared?*’ (0–100, 0 = *never*, 100 = *always*). Reactivity was measured by two questions: 1) ‘*How much did you become more aware of your eating behaviour in the course of the study?*’ (0–100, 0 = *not at all*, 100 = *very much*) and 2) ‘*How much did the morning data assessment influence your eating behaviour on the respective day?*’ (0–100, 0 = *not at all*, 100 = *very much*). Please note that other parts of the dataset have previously been analysed and reported in Reichenberger et al. (2020) [40].

### EMA measures

The present study used signal-contingent sampling: Four times a day (9:00 a.m., 1:00 p.m., 5:00 p.m., and 9:00 p.m.) the app asked participants to report current affective, psychological and physiological states. Participants were prompted by notifications (‘*beeps*’) on their smartphones. All answers were given on a continuous, horizontally presented, rating slider from 0 (= *not at all*) to 100 (= *very much*). The app questionnaires included current positive affects (‘*relaxed’, ‘active’, ‘cheerful’, ‘enthusiastic’, ‘calm’*) as well as negative affects (‘*worried’, ‘depressed’, ‘bored’, ‘nervous/stressed’, ‘irritated’*). Items were partially based on the *Positive and Negative Affect Schedule* (*PANAS* [44]), while additional ones were included in order to cover affects with low thresholds (e.g. *worried*), a high and low arousal space (e.g. *relaxed* as a low-arousal positive affect) as well as study specific contents (e.g. *bored*). In addition to the ‘nervous/stressed’ item, which reflects the momentary state of this feeling, perceived stress *coping* was separately surveyed by two questions worded in the style of the *Perceived Stress Scale* (*PSS* [70]) and adapted to a state level for the purpose of the study: present stress coping: *‘Do you feel that you are on top of things?’*; anticipated stress coping: *‘Do you feel that you can cope with all upcoming things that you will have to do?’*. Although the PSS is generally seen as a measure of stress, it can also be regarded as a two-dimensional model: By means of a confirmatory factor analysis, psychometric research has suggested that the scale involves both reactions to as well as abilities to cope with perceived stressors [71]. For this reason, we chose and adapted items from this scale to assess present as well as anticipated stress coping. Moreover, eating behaviour was assessed similar to previous research [53, 68]: participants were asked to report the extent of their current hunger (*‘How strong is your hunger right now?’*) and food cravings (*‘How strong is your desire to eat certain foods right now?’*). In this context, participants were instructed that ‘certain foods’ refer to certain ‘tasty foods’. In addition to that, participants could fill in up to three eating episodes (i.e. foods that were consumed temporally close together and at the same place) per beep and rate them in terms of the extent to which each eating episode corresponded to their individual eating goal (goal-congruent eating: *‘How much did this eating episode correspond to your eating goal?’*). A mean goal-congruent eating score was calculated for each beep based on a maximum of three reported eating episodes for the respective time interval. By default, the app only allows to submit completely answered EMA questionnaires. However, the goal-congruent eating item(s) could be skipped if participants did not eat between two beeps. For safety reasons (e.g. while driving) as well as practicability, individuals could answer the questions for each of the four beeps with a maximum delay of 1 h after the respective onset. After this period, the prompt to answer a questionnaire disappeared from participants’ smartphones, thus ruling out later entries. In such case, missing values were registered for the respective (missed) time point.

### Network psychometrics

Multilevel vector autoregressive models were estimated using the mlVAR package in R (Version 0.4.2 [72]). Because the number of time points per person was limited, only fixed temporal effects were estimated, as proposed by Bringmann, Lemmens, Huibers, Borsboom, & Tuerlinckx [73]. Additionally, contemporaneous and between-subject networks were estimated. Edges with an arrowhead show the predictive direction of an association between nodes from one time point to the next time point in the temporal network (*x*_*tx*_ → *y*_*tx + 1*_), i.e. in the present study 4 h from *t*_*x*_ to *t*_x *+ 1*_. No prediction was calculated for each day’s last time point on the first time point on the subsequent day. The vector autoregressive model controls for the influence of the previous time point by estimating autoregressive parameters (i.e. how much each node is predicted by its own previous value). Next, the contemporaneous network illustrates partial correlations between residuals so that associations between variables at the same point in time are shown. Lastly, in the between-subject network, edges represent averages of mutual predictions regarding the two connected variables that can be interpreted as cross-sectional partial correlations. Colour-blind friendly designs were used in all network illustrations: Orange nodes belong to the community of eating related nodes, green nodes represent items measuring positive affect while blue ones belong to negative affect items. In addition to that, yellow nodes illustrate stress coping items. Blue edges indicate positive associations whereas red edges represent negative associations. The thickness of every edge illustrates the strength of the respective relationship, which is also specified with coefficients. Only significant edges with effect sizes of *r* > .1 are shown in the network visualisations. Significance (*p* < .05) was determined by multilevel modelling based significance testing through the lme4 package [74]. In the contemporaneous and between-subject networks, at least one of two observed *p*-values must be significant for this edge to be displayed (i.e. ‘or’-rule).

The importance of network nodes is indicated by ‘strength centrality’, the sum of all absolute edge weights. Thus, high strength centrality can reflect a large number of connections to other nodes (highly connected in terms of quantity) and / or strongly weighted edges with other nodes. However, in many cases it is advisable to take negative and positive signs of edges into account. Thus, the centrality measure ‘expected influence’ is calculated by summing the edge weights while keeping their sign. This allows interpretations of variables with negative edge weights [75], for instance, the outgoing expected influence of a resilience variable (e.g. coping capacity) might have a negative sign in a network including harmful behaviours (e.g. overeating). In the temporal network, time-lagging of variables leads to directed associations which allows a distinction between incoming and outgoing strength for each node.

Statistical power considerations are based on a simulation study conducted specifically for mlVAR models that shows that our data set (*N* = 84 participants and 56 time points) is adequate for recovering a network structure of similar size, while maintaining a high specificity (i.e. a low false-positive rate) ([76], pp.107–113). For the present analyses, missing data were not imputed. If incomplete measurements (missing items within a measurement time point) occur, mlVAR can handle them well by using maximum likelihood estimation [77].

## Results

### Descriptive statistics

Participants (72 female, 86.7%), who were mostly university students, had a mean age of 22.7 years (SD = 4.14 years, age range: 18–38 years) and a mean body mass index (BMI) of 21.9 kg/m^2^ (SD = 2.72, range: 17.3–33.1, 6.0% underweight, 84.3% normal weight, 8.4% overweight, 1.2% obese). Please note that participants were also allowed to take part in the study when they aimed at maintaining their body weight (thus not reducing it). None of the participants included in the analyses reported any current or past eating disorder, which was asked for in the beginning of the study. Table 1 illustrates participants’ descriptive statistics based on 3989 data entries (summed repeated measures) of a total number of *N* = 84 participants. In the case of ‘goal-congruent eating’ data are based on 3100 entries (77.7% of all cases) since the amount of reported eating episodes varied, presumably because participants did not always eat something between two assessment time points. Please see also Table 2 in the supplementary materials for zero-order correlations of all included variables.

**Table 1:** Descriptive statistics

Variable	*M*	*SD*	*Median*	*MAD*
*Eating related variables*				
Hunger	25.0	26.1	16	23.7
Food craving	21.5	25.1	12	17.8
Goal-congruent eating	54.6	26.9	57	28.2
*Stress coping*				
Present stress coping	59.8	21.5	63	20.8
Anticipated stress coping	61.0	23.1	65	22.2
*Positive affects*				
Active	34.0	26.4	29	29.7
Cheerful	44.7	27.2	47	34.1
Enthusiastic	24.2	24.3	18	26.7
Relaxed	45.0	26.6	47	31.1
Calm	44.3	26.6	46	32.7
*Negative affects*				
Bored	15.2	20.0	8	11.9
Depressed	11.0	18.3	0	0
Irritated	11.7	17.7	4	5.9
Nervous/stressed	21.3	21.9	15	22.2
Worried	17.8	20.8	11	16.3

*Note. N* = 84. Range of all items = 0–100. For complete item texts, please see the ‘Methods’ (EMA measures) section of the manuscript. *M* Mean, *SD* Standard deviation, *MAD* Median absolute deviation

### Compliance and reactivity

The average compliance rate over all participants included in the network analysis was 84.8%. Moreover, participants estimated the extent of purposefully shortening EMA questionnaires rather low (*M* = 7.97, *SD* = 10.78, *N* = 79). Additionally, reactivity in terms of becoming more aware of their eating behaviour was moderate (*M* = 54.94, *SD* = 24.75, *N* = 79). Participants also reported low to moderate reactivity concerning the influence of the morning assessments on the eating behaviour on the respective days (*M* = 27.22, *SD* = 25.82, *N* = 79).

### Temporal, contemporaneous and between-subject networks

**Fig. 2:**
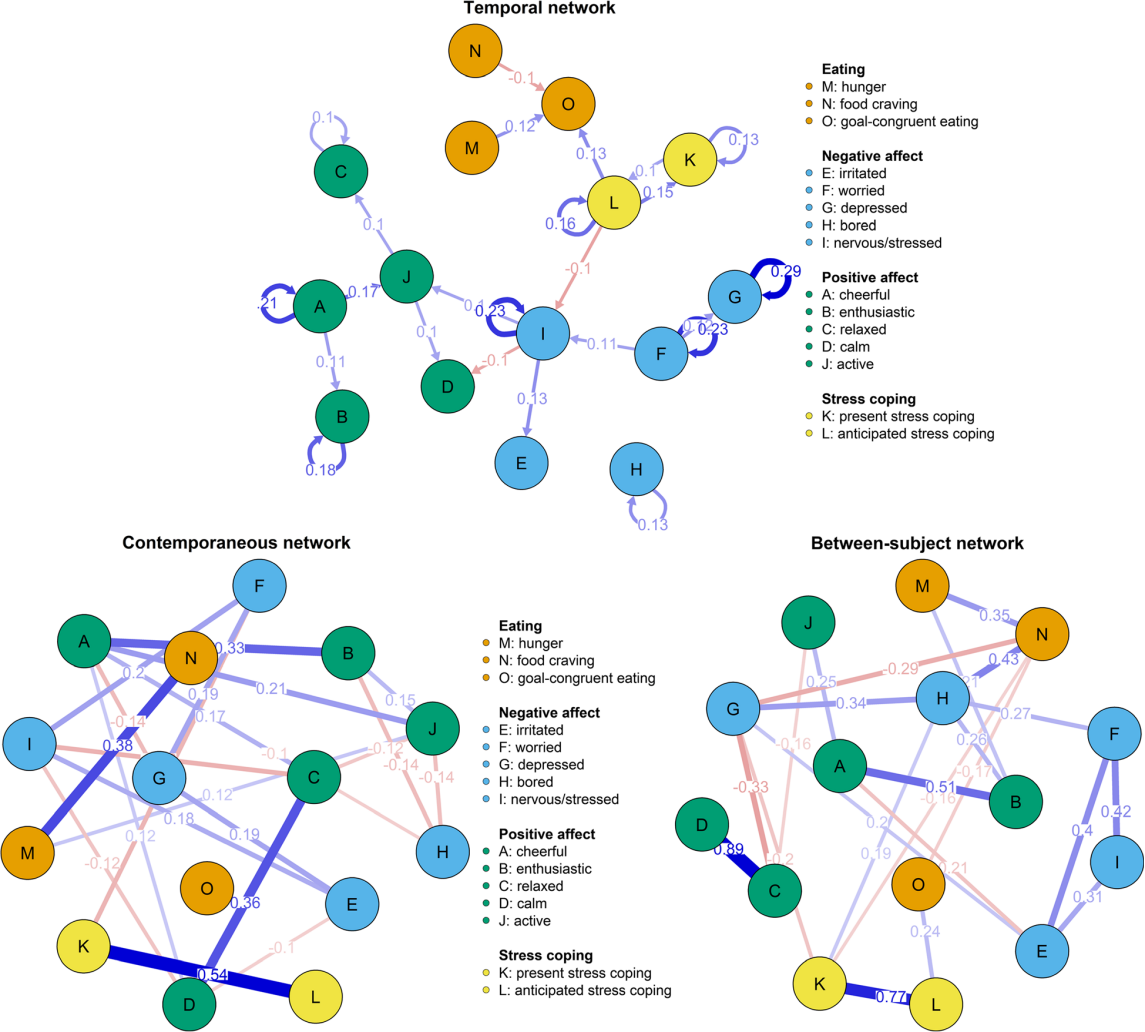
Temporal, contemporaneous and between-subject networks. **Note**. Please see the legend above for meanings of coloured circles as well as abbreviations. Only significant associations that show effect sizes of *r* > .1 are displayed in the networks. Blue edges indicate a positive association, whereas red edges indicate a negative one. The coefficients are displayed on or next to the respective edges. Please see also Fig. 5 in the supplementary materials, which includes all associations (*r* > .1) regardless of significance level

**Fig. 3:**
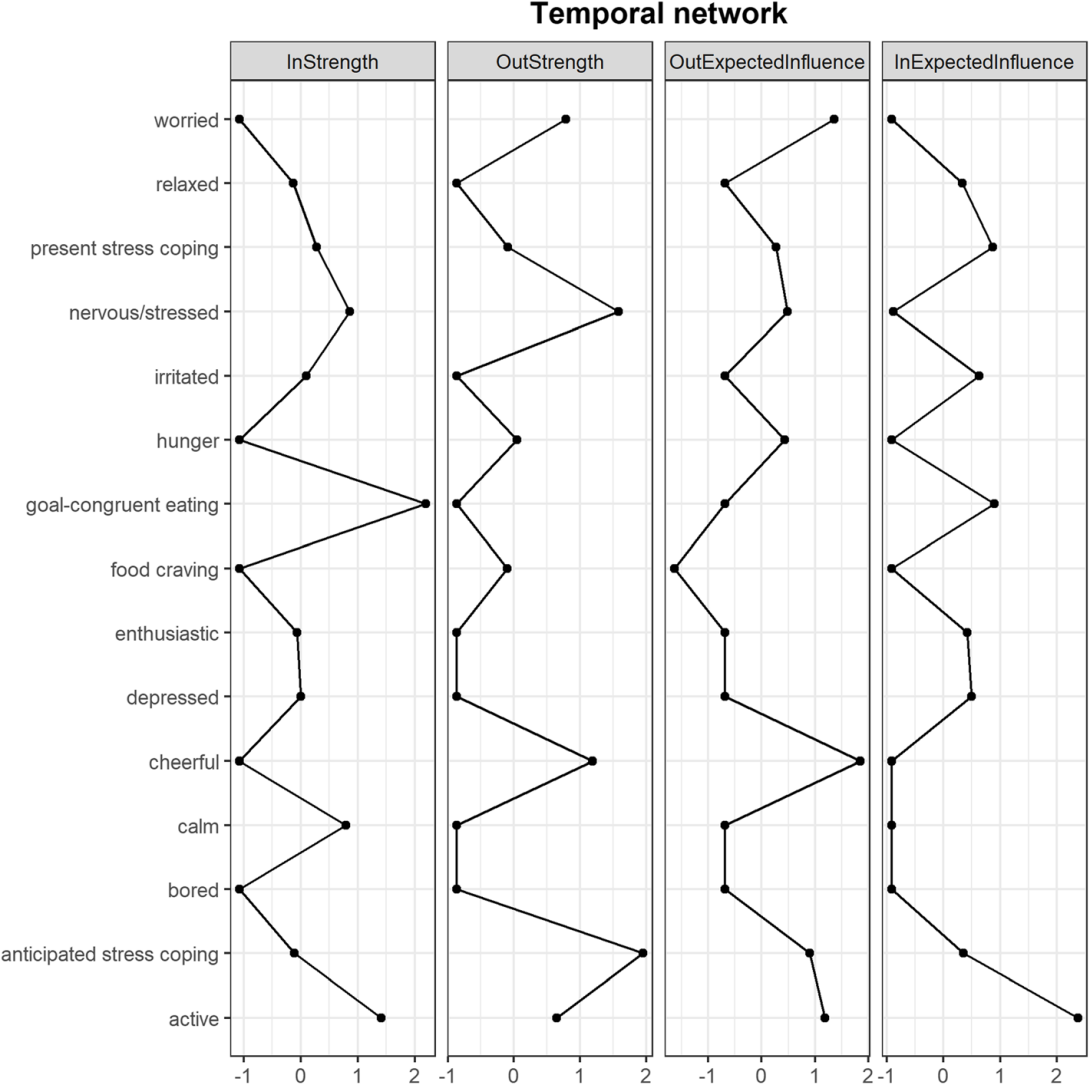
Z-scaled incoming and outgoing strength centralities as well as expected influences in the temporal network. **Note**. Connecting lines are merely for visualisation purposes

**Fig. 4:**
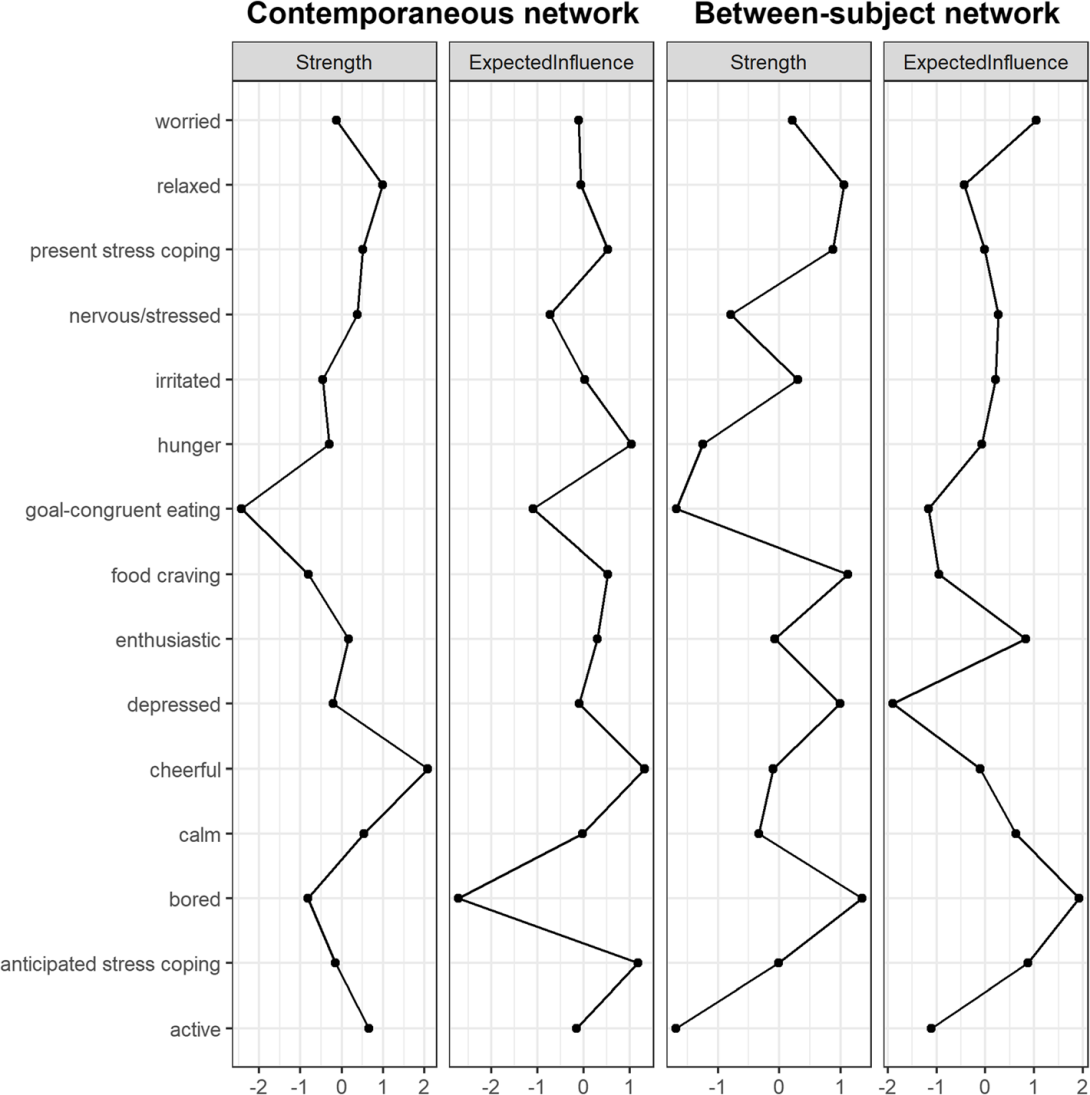
Z-scaled strength centralities and expected influences in the contemporaneous network as well the between-subject network. **Note**. Connecting lines are merely for visualisation purposes

The results of the three network analyses are shown in Fig. 2. Moreover, the results of the analyses of strength centrality for all networks are displayed in Figs. 3 and 4. A selection of significant results that might be most important for eating behaviour research will be reported in more detail below.

### Temporal network

The node ‘goal-congruent eating’ showed higher incoming than outgoing expected influence, thus being rather an outcome variable than a predictor that influences subsequent states. It was influenced by three nodes: Food cravings negatively predicted subsequent goal-congruent eating (*r* = −.103, *SE* = .028, *p* < .001), while hunger positively predicted it (*r* = .122, *SE* = .026, *p* < .001). Furthermore, goal-congruent eating was also positively predicted by the preceding anticipated stress coping *(r* = .129, *SE* = .055, *p* = .020). However, this was neither the case for stress experience nor for any of the affects. Lastly, both anticipated stress coping (*r* = .164, *SE* = .035, *p* < .001) as well as present stress coping (*r* = .135, *SE* = .038, *p* < .001) were auto-correlated, indicative of temporal stability. Additionally, present stress coping predicted later anticipated stress coping (*r* = .104, *SE* = .034, *p* = .002) and vice versa (*r* = .146, *SE* = .038, *p* < .001).

### Contemporaneous network

Higher present stress coping co-occurred with higher anticipated stress coping (*r* = .538, *p*_*1 → 2*_ < .001, *p*_*2 → 1*_ < .001) and with lower worry (*r* = −.156, *p*_*1 → 2*_ < .001, *p*_*2 → 1*_ < .001). Moreover, hunger co-occurred with food cravings (*r* = .384, *p*_*1 → 2*_ < .001, *p*_*2 → 1*_ < .001). No contemporaneous associations of goal-congruent eating were found, which was completely isolated in the contemporaneous network.

### Between-subject network

Individuals with higher present stress coping also anticipated high stress coping (*r* = .768, *p*_*1 → 2*_ < .001, *p*_*2 → 1*_ < .001). Similar to the temporal network, participants with higher anticipated stress coping also reported higher goal-congruent eating behaviour (*r* = .240, *p*_*1 → 2*_ = .064, *p*_*2 → 1*_ = .021). Again, also on the between-subject level, food cravings were positively related to hunger (*r* = .353, *p*_*1 → 2*_ *=* .002, *p*_*2 → 1*_ < .001), however, they were also related to feeling bored (*r* = .433, *p*_*1 → 2*_ *=* .003, *p*_*2 → 1*_ < .001).

## Discussion

The present study aimed at improving the knowledge about stress, affect, and eating related variables as well as their dynamic interplay through employing psychological networks of EMA data gathered across 14 days.

### Goal-congruent eating behaviour and stress

While no significant outgoing strength was found for ‘goal-congruent eating’, its incoming strength suggests defining it as an outcome (dependent) variable, both in terms of absolute incoming strength and also in consideration of the sign of the coefficients. Goal-congruent eating reflects the degree to which recently consumed meals are congruent with individuals’ eating goals and thus ‘adapts’ to the individuals’ preferred diet or food composition. In that sense, goal-congruent eating is similar to diet adherence, which can be assessed for any diet and is related to successful weight management [8, 78].

Higher goal-congruent eating was preceded by higher anticipated stress coping, while relationships were neither found for momentary feelings of stress, nor for present stress coping. This finding dovetails with research showing that low confidence in one’s own coping abilities results in more problematic eating behaviours [79]. Stress that is not buffered by coping strategies might lower self-control and thus compromise goal-congruent eating [20]. The reason why anticipated stress coping was predictive of eating and not current stress experience or current stress coping might have to do with the time window covered: participants might have mentally ‘time travelled’ through the upcoming 4 h, investigating any potential stressors and evaluating their coping capacity. These 4 h corresponded to the time window during which subsequent meals occurred that were covered by the goal-congruent eating item, thus temporal correspondence was high. Relationships between stress and eating behaviour might be fleeting and short lived or delayed, as revealed by previously reported day level retrospective analyses that showed different results intra-day vs. on day level [40]. Stress experience and coping at time point t_x_ were not predictive of goal-congruent eating at timepoint t_x + 1_ possibly because both reflect momentary or recent experiences and neither entail outlook. However, when the specific temporal resolution was extended or aggregated to a daily level, previous research reported associations between stress and eating behaviour [40, 80], which was measured in terms of food categories (e.g. vegetables), respectively, the *amount* of consumed foods in these studies. Worthy of note, networks show associations after controlling for all other variables’ potential influence, which does not exclude the possibility that momentary stress might predict goal-congruent eating when considered as a single predictor. Moreover, the between-subject network mirrored the importance of stress coping: across the whole assessment period, individuals who often reported a high anticipated stress coping capacity, often also showed more goal-congruent eating behaviour.

In sum, the reported association suggests a potential point of intervention: increasing individuals’ general (i.e. behaviour unspecific) stress coping capacities / efficacies (as were assessed in the present study) may support them to achieve dieting success by matching their personal eating goal more closely. Previous research concluded similarly that behavioural stress coping interventions might help reduce obesity and support healthy eating behaviours [81]. Having a high stress coping capacity based on, for instance, approach-orientated (rather than avoidant-orientated) coping strategies, might help individuals to stick to their dieting plans and pursue their healthy eating goals [82]. Furthermore, the prospective nature of the observed relationship would allow interventions triggered by low ratings of anticipated stress coping (i.e. Just-In-Time Adaptive Interventions). In such a case, participants would then have time to take preventive measures, such as removing tempting foods or going for a relaxing walk.

### Stress dynamics

The results support a distinction between momentary ratings of experiencing stress (node *I*) and subjective coping with stress (nodes *K* and *L*) as these were unrelated in the contemporaneous and the between-subject networks. In fact, only in the temporal network did they relate to each other: the higher the anticipated stress coping, the lower the subsequent feeling of stress, but not vice versa. This can be seen as further proof that stress experience and the mobilisation of resources are two, partially independent processes, yet with potential temporal relationships. The direction of associations suggests that anticipated coping might be the driving force of this relationship, again pointing to coping as a useful leverage point for interventions. Importantly, in all three networks, associations between both specific measures of stress coping were found.

Firstly, we found a positive feedback loop between present and anticipated stress coping: Both coping capacities showed auto-correlations, however, both also predicted the other variable at the following time point. Feeling capable of coping with current tasks positively influenced the subsequent feeling of being able to cope with upcoming tasks or problems, respectively vice versa. This finding represents a certain stability of stress coping capacities and a positive upward loop. By implication, this might even boost the interventional potential regarding goal-congruent eating behaviour (see ‘*Goal-congruent eating behaviour and stress’)*, as the latter might benefit from multiple stress coping strategies that could reinforce themselves over time. Secondly, these results go in line with the results of the contemporaneous network, where both coping capacities were moderately associated at the same time point, i.e. feeling capable of coping with stress at one moment was positively associated with feeling able to cope with upcoming stressors. Thirdly, in the between-subject network, present and anticipated stress coping were strongly associated: Participants who often reported high present stress coping, often also tended to anticipate high stress coping. This appears to be in line with views of coping as a mostly dispositional and stable construct [83]. To conclude, both stress coping capacities appear to be meaningfully related and thus clustered together in all three networks.

### Goal-congruent eating behaviour and affects

As already discussed, no relationship between *feeling* stressed (node *I*) and goal-congruent eating (node *O*) was found. Neither was this the case for experiencing negative or positive affects: no direct associations were found between affects and goal-congruent eating behaviour in either network. These results tap into the controversy around emotional eating [37, 52, 55, 56], in the course of which several studies, reviews and meta-analyses came to different conclusions about what this relationship is and who might show it. Yet, note that according to the individual difference model of emotional eating, the direction of this relationship depends on emotional eating traits [46, 48, 53]. Such analyses, in which a continuous between-subject variable (e.g. emotional eating trait) moderates the within-subject relationship were not conducted in the present study, so positive and negative associations might have cancelled out. For the reverse direction, one could have expected that ‘diet lapses’ (i.e. low scores on ‘goal-congruent eating’) increase subsequent negative and decrease subsequent positive affects. Yet, participants’ eating behaviour did not influence subsequent affects. Such results contrast with previous laboratory research that for instance found that eating chocolate can cause positive affects such as joy as well as negative affects such as guilt [59]. Although such results were not observed in our mostly healthy weight sample, effects like these might be potentiated in individuals with overweight/obesity or specific eating disorders (e.g. bulimia nervosa or binge eating disorder), who might experience strong weight concerns.

As for future directions, following the distinction between *experiencing* stress and *coping with* stress (see above), it might be worthwhile differentiating *experiencing* affects and *dealing / coping with* affects. Thus, effects on eating behaviour should potentially be differentiated in terms of a) relationships of affect itself (i.e. the sole occurrence of an affective state) with subsequent behaviour vs. b) relationships of reactions to affects (e.g. appraisal or emotion regulation) and subsequent behaviour. Similarly to stress (discussed above), it appears conceivable that feeling, for instance, angry does not always influence eating behaviours, because specific subsequent appraisals or emotion regulating reactions might be influential for subsequent behaviour. Indeed, research has shown that emotion regulation can play an influential role in eating behaviour [84]. Additionally, emotion-orientated coping and avoidance distraction have previously been found to be related to emotional eating on a cross-sectional level while controlling for negative affect, which itself did not show a significant and unique association with emotional eating [41]. Such results might question a strong or direct link between negative affect and emotional eating and suggest the role of intermediate variables.

### Homeostatic and hedonic eating behaviours

The present network analyses might also refer to the long ‘mind vs. metabolism’ debate [85] concerning the difference between homeostatic hunger and hedonic food craving. This distinction between two highly correlated constructs appears reasonable, as both variables had differential impacts on eating behaviours in the present study: Food cravings negatively predicted subsequent goal-congruent eating in the temporal network. In line with dysfunctional interpretations, food cravings have previously been found to be related to decreased dieting success [23]. In particular, cravings might lead to consumption of foods that do not correspond with one’s healthy eating goal such as chocolate or fast foods [19, 27], particularly in the afternoon [68]. On the other hand, despite showing largely similar time courses across the day [68], hunger seemed to trigger the intake of rather goal-congruent foods as shown by a positive association in the present study. Therefore, results suggest that eating because of hunger (i.e. a homeostatic behaviour) shows a positive effect on goal-congruent food consumption in contrast to a rather negative influence of food cravings (i.e. a hedonic behaviour). By implication, this assumption could go in line with eating in the absence of hunger as a risk factor for high energy intake [86]. In contrast, eating in response to hunger is considered functional by most ‘intuitive eating’ inspired diets, which might have effects on weight maintenance [87], however, evidence for a general health beneficial potential of ‘intuitive eating’ concepts is still needed [88]. Importantly, distinguishing hunger from craving might be difficult for individuals: in the present study, experiences of hunger and cravings co-occurred simultaneously within individuals (contemporaneous network) as well as co-occurred across participants (between-subject network). Such results correspond to findings of concurrently experiencing (or at least reporting) food cravings and hunger [68]. Therefore, it might be difficult for individuals to differentiate between hunger and craving, especially when hunger mirrors craving around mealtimes [68]. Thus, training individuals to distinguish hedonic cravings from homeostatic hunger seems a useful approach in terms of supporting healthy eating.

Lastly, the cross-sectional relationship of higher *boredom* going along with higher food cravings corresponds to findings showing that food cravers experience increased boredom throughout the day compared to non-cravers [89]. Moreover, it has been shown that boredom-prone individuals reported to eat because of negative emotions [90] which could constitute a cross-sectional link between trait-like boredom proneness and eating as form of emotion regulation (see ‘*Goal-congruent eating behaviour and affects’).* As ‘bored’ showed the highest expected influence in the present between-subject network, it might therefore be meaningful to be assessed and examined in future affective behavioural research. However, being bored did not show any positive correlation with other affect items a priori grouped as ‘negative’ in the contemporaneous network. Thus, this may suggest that including ‘bored’ as a component of (mean) negative affect scores could be problematic in populations such as the present one.

### Strengths, limitations, and future research

Self-reported EMA data are always subjective and thus might to some degree reflect the self-presentation of our participants – although, it must be noted that there is yet no established objective measure of affect and stress coping in everyday life. Thus, we selected and adapted measures of affect and state coping that were taken from larger scales (PANAS, respectively, PSS) as well as single, distinct items, as is common practice in EMA research [91]. Thus, results could potentially differ from studies using complete (and validated) scales and different analytic approaches. We assessed general coping efficacies, but did not differentiate specific coping styles (such as approach- vs. avoidant-orientated), which would be fruitful, but would also have to be balanced against participant burden that is driven by a high number of queries. Similarly, we did not differentiate food environments, which could however influence the availability of foods matching individual eating goals. Regarding generalisability, it has to be taken into account that our sample consisted of mostly young, predominantly academic and female participants (with limited age range and mostly normal weight), which is not representative for the general population. Nevertheless, we consider it a strength of the present study that we explicitly focussed on diet-interested individuals which enables the investigation of goal-congruent eating behaviour. Additionally, the observed associations might vary between different desired diet outcomes (for instance, maintaining vs. reducing body weight), which were not differentiated in the present study and could be taken into account in future studies. To our knowledge, the present study is the first one using network analysis to explore goal-congruent eating behaviour in diet-interested individuals. This approach allows investigations of temporal sequence and simultaneity of relationships as well as cross-sectional associations. Regarding sample size and statistical power, the present exploratory study shows acceptable parameters [76], however, future studies aiming to replicate our findings should increase the sample size and the number of time points at which data are gathered (more repeated measures). Moderate sample sizes such as ours can result in reduced sensitivity (not recovering all edges that could have been included with a larger sample size).

## Conclusions

Two key findings of the present study should be highlighted: Regarding methodology, eating behaviour research can clearly benefit from EMA-based psychological networks, since they uncover multidirectional and time-lagged relationships of affects, stress, and health relevant behaviours among others. Conceptually, we showed that stress *coping* might play an important role for goal-congruent eating behaviour in young (female) adults who try to manage their weight. Of note, experiencing stress or negative as well as positive affects were not directly related to goal-congruent eating, calling for more research on potential moderators and mediators of these relationships. Our study aimed to pave the way for future EMA and ecological momentary intervention (EMI) studies in the field of eating behaviour or eating disorders that focus on unique associations between such variables. Given the rise of new methods such as EMI [92–94] or Just-In-Time Adaptive Interventions [95, 96] in smartphone-supported eating behaviour research, we recommend network analyses, as they help to gain knowledge about multidirectional associations and might also point to potentially relevant intervention targets.

## Supplementary Information

**Additional file 1: Fig. 5**. Temporal, contemporaneous and between-subject networks without significance threshold.

**Additional file 2: Table 2**: Means, standard deviations, and zero-order correlations with confidence intervals.

**Additional file 3:** Information regarding the present empirical study.

## Data Availability

The datasets used and/or analysed during the current study are available from the corresponding author on reasonable request.
