# Supplementary material for: Networks of stress, affect and eating behaviour: anticipated stress coping predicts goal-congruent eating in young adults
Source: Int J Behav Nutr Phys Act. 2021 Jan 9;18:9. doi: 10.1186/s12966-020-01066-8 (PMC7796605; doi:10.1186/s12966-020-01066-8)
Supplement: Supplementary file 1 — Additional file 1: Fig. 5. Temporal, contemporaneous and between-subject networks without significance threshold. [file 12966_2020_1066_MOESM1_ESM.pdf]

Networks of stress, affect and eating behaviour: anticipated stress coping predicts goal-congruent eating in young adults
Björn Pannicke, Tim Kaiser, Julia Reichenberger and Jens Blechert
International Journal of Behavioral Nutrition and Physical Activity
https://doi.org/10.1186/s12966-020-01066-8
Supplementary material: Figure 5
Temporal, contemporaneous and between-subject networks without significance threshold

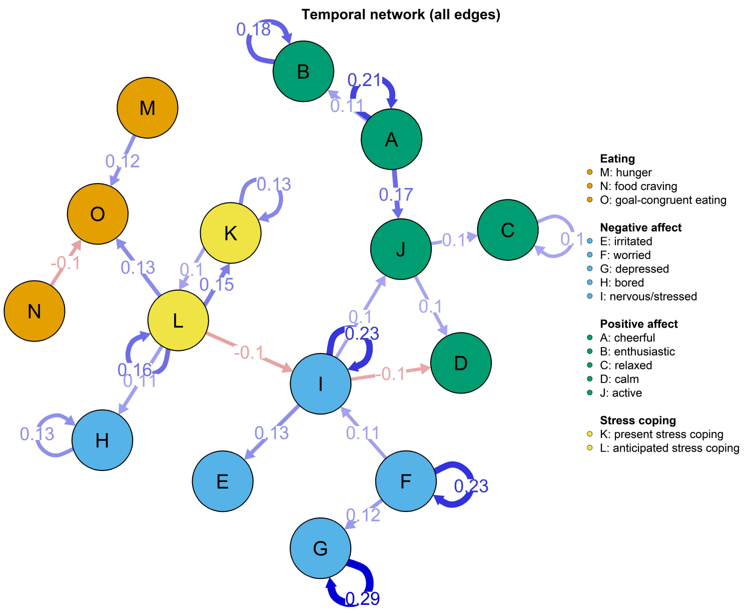

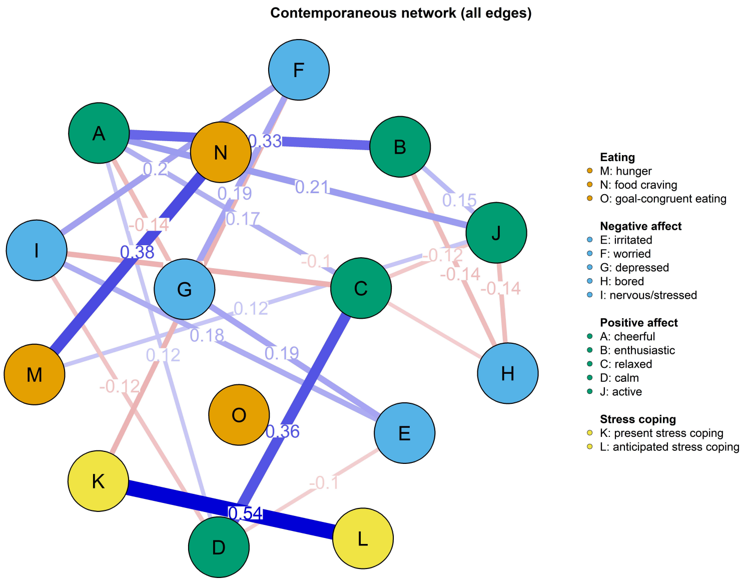

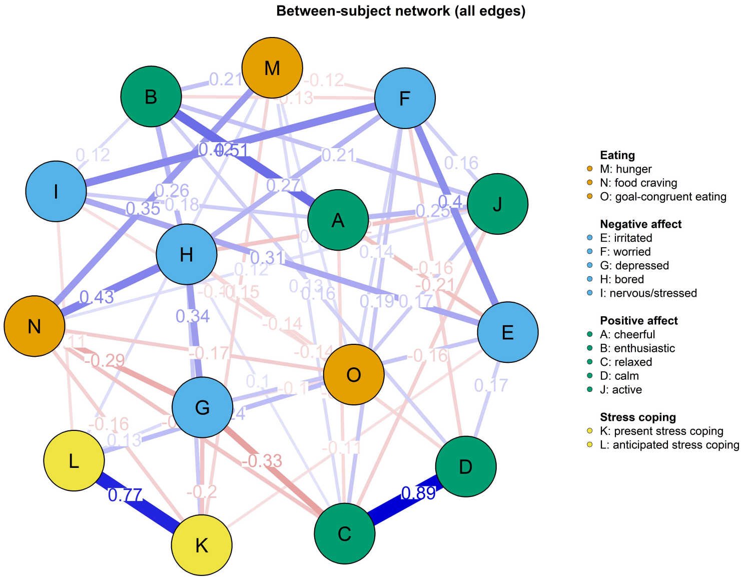

Figure 5. Temporal, contemporaneous and between-subject networks without significance threshold. Note. Please see the legend above for meanings of coloured circles as well as abbreviations. All associations that show effect sizes of r > .1 are displayed in the networks. Blue edges indicate a positive association, whereas red edges indicate a negative one. The coefficients are displayed on or next to the respective edges
