# Supplementary material for: Networks of stress, affect and eating behaviour: anticipated stress coping predicts goal-congruent eating in young adults
Source: Int J Behav Nutr Phys Act. 2021 Jan 9;18:9. doi: 10.1186/s12966-020-01066-8 (PMC7796605; doi:10.1186/s12966-020-01066-8)
Supplement: Supplementary file 2 — Additional file 2: Table 2: Means, standard deviations, and zero-order correlations with confidence intervals. [file 12966_2020_1066_MOESM2_ESM.pdf]

Networks of stress, affect and eating behaviour: anticipated stress coping predicts goal-congruent eating in young adults

Björn Pannicke, Tim Kaiser, Julia Reichenberger and Jens Blechert

International Journal of Behavioral Nutrition and Physical Activity
https://doi.org/10.1186/s12966-020-01066-8

Supplementary material: Table 2

Means, standard deviations, and zero-order correlations with confidence intervals

Variable	*M*	*SD*	1	2	3	4	5	6	7	8	9	10	11	12	13	14
1. Cheerful	44.70	27.18														
2. Enthusiastic	24.16	24.28	.61**<br>[.59, .63]													
3. Relaxed	44.97	26.55	.51**<br>[.49, .53]	.34**<br>[.31, .37]												
4. Calm	44.33	26.64	.55**<br>[.53, .57]	.39**<br>[.36, .41]	.73**<br>[.71, .74]											
5. Irritated	11.67	17.69	-.21**<br>[-.24, -.18]	-.07**<br>[-.10, -.04]	-.25**<br>[-.27, -.22]	-.26**<br>[-.29, -.23]										
6. Worried	17.79	20.80	-.22**<br>[-.25, -.19]	-.09**<br>[-.12, -.06]	-.29**<br>[-.32, -.27]	-.32**<br>[-.35, -.29]	.46**<br>[.43, .48]									
7. Depressed	11.00	18.27	-.33**<br>[-.36, -.30]	-.17**<br>[-.20, -.14]	-.32**<br>[-.35, -.30]	-.32**<br>[-.35, -.30]	.43**<br>[.41, .46]	.48**<br>[.46, .51]								
8. Bored	15.21	19.90	-.13**<br>[-.16, -.10]	-.08**<br>[-.11, -.05]	-.05**<br>[-.08, -.02]	-.05**<br>[-.08, -.02]	.16**<br>[.13, .19]	.15**<br>[.11, .18]	.19**<br>[.16, .22]							
9. Nervous/stressed	21.34	21.87	-.19**<br>[-.22, -.16]	-.06**<br>[-.09, -.03]	-.39**<br>[-.41, -.36]	-.38**<br>[-.40, -.35]	.47**<br>[.45, .50]	.56**<br>[.54, .58]	.36**<br>[.33, .38]	.07**<br>[.04, .10]						
10. Active	34.02	26.38	.51**<br>[.48, .53]	.46**<br>[.43, .48]	.20**<br>[.17, .23]	.28**<br>[.25, .31]	-.04*<br>[-.07, -.01]	-.07**<br>[-.10, -.03]	-.17**<br>[-.20, -.14]	-.16**<br>[-.19, -.13]	.02<br>[-.01, .05]					
11. Present stress coping	59.77	21.54	.48**<br>[.46, .50]	.35**<br>[.32, .38]	.50**<br>[.48, .52]	.53**<br>[.50, .55]	-.30**<br>[-.33, -.27]	-.43**<br>[-.46, -.41]	-.44**<br>[-.46, -.41]	-.06**<br>[-.09, -.02]	-.35**<br>[-.38, -.33]	.28**<br>[.25, .31]				
12. Anticipated stress coping	61.00	23.05	.49**<br>[.47, .52]	.36**<br>[.33, .39]	.48**<br>[.46, .51]	.52**<br>[.50, .55]	-.28**<br>[-.31, -.25]	-.40**<br>[-.43, -.38]	-.41**<br>[-.43, -.38]	-.07**<br>[-.10, -.04]	-.33**<br>[-.36, -.30]	.32**<br>[.29, .35]	.84**<br>[.83, .85]			
13. Hunger	25.04	26.10	.07**<br>[.03, .10]	.05**<br>[.02, .08]	.02<br>[-.01, .05]	.02<br>[-.01, .05]	.05**<br>[.02, .08]	-.02<br>[-.05, .01]	-.04**<br>[-.07, -.01]	.03<br>[-.00, .06]	.02<br>[-.01, .05]	.07**<br>[.04, .10]	.05**<br>[.02, .08]	.06**<br>[.03, .09]		
14. Food Craving	21.53	25.11	.06**<br>[.03, .09]	.08**<br>[.05, .11]	.01<br>[-.02, .04]	.01<br>[-.02, .04]	.11**<br>[.08, .14]	.05**<br>[.02, .08]	.00<br>[-.03, .03]	.15**<br>[.12, .18]	.07**<br>[.04, .10]	.04**<br>[.01, .07]	.02<br>[-.01, .05]	.02<br>[-.01, .05]	.43**<br>[.41, .46]	
15. Goal-congruent eating	54.61	26.86	.17**<br>[.13, .20]	.13**<br>[.09, .16]	.11**<br>[.08, .15]	.16**<br>[.12, .19]	-.07**<br>[-.10, -.03]	-.05**<br>[-.08, -.01]	-.15**<br>[-.19, -.12]	-.09**<br>[-.12, -.05]	-.05**<br>[-.09, -.02]	.19**<br>[.16, .23]	.23**<br>[.19, .26]	.26**<br>[.22, .29]	-.01<br>[-.04, .03]	-.06**<br>[-.10, -.03]

Note. M = mean and *SD* = standard deviation. Values in square brackets indicate the 95% confidence interval for each correlation. * indicates $p < .05$. ** indicates $p < .01$.
