# Supplementary material for: Networks of stress, affect and eating behaviour: anticipated stress coping predicts goal-congruent eating in young adults
Source: Int J Behav Nutr Phys Act. 2021 Jan 9;18:9. doi: 10.1186/s12966-020-01066-8 (PMC7796605; doi:10.1186/s12966-020-01066-8)
Supplement: Supplementary file 3 — Additional file 3: Information regarding the present empirical study. [file 12966_2020_1066_MOESM3_ESM.pdf]

Networks of stress, affect and eating behaviour: anticipated stress coping predicts goal-congruent eating in young adults

Björn Pannicke, Tim Kaiser, Julia Reichenberger and Jens Blechert (2020)

International Journal of Behavioral Nutrition and Physical Activity

https://doi.org/10.1186/s12966-020-01066-8

Supplementary material: Information regarding the present empirical study

Diet-interested individuals were recruited by means of a study announcement via e-mail and by word of mouth at several universities across Austria and Germany. Individuals with the intention to 'maintain or reduce their body weight' were invited to participate in the study, as this ensured that participants followed specific eating goals. This information can be found in the 'Participants' part of the 'Methods' section in our manuscript.

The sample consisted of mostly female, young adults who managed their weight. Thus, the sample has a limited generalisability regarding the target group of all dieting adults. We acknowledged this information in the 'Limitations and future research' part of the 'Discussion' section in our manuscript.

Participants who had more than 50 % missing data (i.e. less than 28 answered app-questionnaires) were excluded from the analyses. This was the case for 6 participants. Before the study, we set the threshold to ensure sufficient repeated measures per person. Missing data were not imputed in the analyses.
